# Edge Effects and Pitfall Trap Design Influence Spider Diversity and Assemblages in Canola Agroecosystems on the Canadian Prairies

**DOI:** 10.1002/ece3.72205

**Published:** 2025-09-23

**Authors:** Kirra Kent, Aldo Ríos Martínez, Kristen Guelly, Jaime Pinzon, Boyd A. Mori

**Affiliations:** ^1^ Department of Agricultural, Food and Nutritional Science, 4‐10 Agriculture/Forestry Centre University of Alberta Edmonton Alberta Canada; ^2^ Canadian Forest Service Natural Resources Canada Edmonton Alberta Canada

**Keywords:** edge effects, Linyphiidae, Lycosidae, passive sampling

## Abstract

Spiders (Araneae) are generalist predators in agroecosystems and may contribute to biological control in canola ( *Brassica napus* L. and *B. rapa* L.). However, their diversity and community structure remain understudied in the Canadian Prairies. To address this knowledge gap, we surveyed spider assemblages in the Aspen Parkland region of Alberta, Canada, using pitfall traps placed at field edges and interiors during the 2021 and 2022 growing seasons. We collected 968 spiders in 74 species across 14 families, with Lycosidae and Linyphiidae being the most abundant. Spider abundance was consistently greater at field edges, although family‐level composition varied by year and location. In 2023, we tested pitfall trap modifications to improve spider retention, evaluating (1) trap diameter, (2) preservative substrate (glass beads vs. propylene glycol), and (3) polytetrafluoroethylene (PTFE; Fluon, a non‐stick fluoropolymer) coated trap interiors (to reduce friction and prevent escape). This was tested across canola habitats adjacent to grass‐ or tree‐dominated non‐crop habitat edges. Trap catches, and resulting richness and diversity, were significantly higher in large PTFE‐treated traps, particularly in treed‐edge habitats compared to other treatment combinations. These results underscore the importance of non‐crop field margins in maintaining spider diversity and highlight design improvements to optimize passive sampling. Our findings provide a regional baseline for spider assemblages in canola systems and offer methodological advances to support future ecological monitoring and conservation biological control efforts in Prairie agroecosystems.

## Graphical abstract

Spiders are important generalist predators in canola agroecosystems, but their diversity remains poorly studied on the Canadian Prairies. We surveyed spider assemblages in the Aspen Parkland region and tested pitfall trap modifications to improve sampling. Our results highlight the importance of field edges for spider diversity and offer improved methods for monitoring in Prairie agroecosystems.

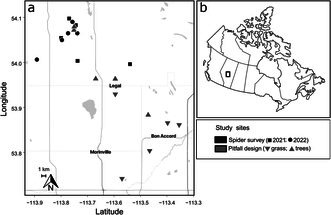

## 1 Introduction

Agroecosystems have significantly altered landscapes, creating a patchwork of disturbed (e.g., cultivated fields) and relatively undisturbed semi‐natural habitats. These semi‐natural areas, such as grasslands, woodlands, and wetlands, although influenced by nearby agricultural activity, offer relatively stable environmental conditions and alternative food sources, functioning as important refugia for biodiversity (Duflot et al. 2014; Bartual et al. 2019). This landscape heterogeneity gives rise to transitional boundary zones, commonly referred to as “edges”, where two habitat types meet. Edges are often seen as beneficial to species inhabiting agricultural habitats but may act as ecological filters or barriers for species associated with more natural or semi‐natural environments. Variation in neighboring semi‐natural habitats (e.g., trees, shrubs, and grass) can shape the vegetation and microclimate at the field edge, which can influence the composition and activity of predators (Perrot et al. 2021; Holland et al. 2016). These habitat‐driven differences can affect predator colonization and their potential for pest suppression within the crop. The interactions between adjacent habitats and their boundaries, referred to as edge effects, can significantly alter local biodiversity relative to adjacent habitats (Dauber and Wolters 2004). Understanding edge effects is critical for the sustainable management of agroecosystems, with potential benefits for both biodiversity conservation and agricultural productivity (Gallé et al. 2020).

Canola (oilseed rape) ( *Brassica napus* L. and *B. rapa* L., Brassicaceae) is one of the most widely cultivated oilseed crops globally, valued primarily for its use in animal feed, edible vegetable oil production, and as a biofuel feedstock. In Canada, canola occupies over 8.7 million hectares, predominantly across the Prairie Provinces, making it the second‐largest crop by area (Statistics Canada 2025). Canola contributes significantly to the Canadian economy, with an estimated economic impact of CAD$43 billion annually (Global Data 2024). Despite its economic importance, canola production is challenged by various biotic stressors, including arthropod pests, weeds, and plant pathogens (FAO 2022). Key arthropod pests in North American canola systems include flea beetles (*Phyllotreta* spp., Coleoptera: Chrysomelidae), diamondback moths (*Plutella xylostella* (L.), Lepidoptera: Plutellidae), bertha armyworms ( *Mamestra configurata* Walker, Lepidoptera: Noctuidae), and lygus bugs (*Lygus* spp., Hemiptera: Miridae) (Dosdall and Mason 2010). While insecticides are commonly used for pest control, they carry environmental costs and may disrupt beneficial insect populations (Ansari et al. 2014; Sharma et al. 2019). As a result, conservation biological control, a sustainable pest management approach that integrates and enhances the activity of naturally occurring predators (Begg et al. 2017), is becoming increasingly important. Generally, the role of spiders in insect pest management remains largely understudied in Canada, particularly in canola cropping systems (Furlong et al. 2008; Sarwar 2013; Benamú et al. 2017).

Spiders, especially epigaeic (ground‐dwelling) taxa, are among the most abundant and ecologically significant arthropod predators in agroecosystems. They contribute to pest suppression through direct predation (Nyffeler and Benz 1987; Alderweireldt 1994; Carter and Rypstra 1995; Benamú et al. 2017) and via non‐consumptive effects, such as deterring herbivores through the presence of webs, silk, exuviae, or frass (i.e., excrement) (Michalko et al. 2019). Recent studies suggest that spiders may help manage aphid (Hemiptera: Aphididae), flea beetle, and diamondback moth populations in canola (Quan et al. 2011; Ekbom et al. 2014; Amjad et al. 2017; Farias et al. 2021; Elliott et al. 2023). Although many spiders are generalist predators, their habitat preferences and hunting strategies vary widely (Cardoso et al. 2011). Consequently, spiders are highly responsive to edge effects in agroecosystems (Prieto‐Benítez and Méndez 2011; Baba et al. 2018), and the variability of spider community composition between habitat types may result in differential pest control potential.

Spiders are frequently sampled using passive methods such as pitfall trapping, which is well‐suited for monitoring ground‐active arthropods and their potential for biological control. Passive sampling involves the use of traps to collect insects and returning later to retrieve the specimens, but this method is subject to activity‐density bias, favoring the capture of larger and more mobile organisms (Zou et al. 2012; Engel et al. 2017). Pitfall traps may be installed either as kill traps, using preservatives like propylene glycol, or as live traps to enable bioassays, reduce bycatch mortality, or preserve specimens for molecular work. Live trapping is especially advantageous when targeting DNA‐quality samples or sensitive taxa, though retaining live spiders can be challenging due to their escape behavior and in‐trap predation (Greenslade 1964; Joosse and Kapteijn 1968). Live‐passive collection remains a limitation for certain spider taxa, particularly in the case of pitfall traps, which have been shown to be less effective in this regard compared to their use as kill traps (Weeks Jr. and McIntyre 1997).

This study aims to document spider diversity and community structure in canola‐dominated agroecosystems in the Aspen Parkland region of Alberta, Canada. Specifically, this study evaluates the abundance, diversity, and richness of spider communities in canola agroecosystems between field interiors and edges. Field edges, which are located closer to areas (e.g., surrounding non‐crop vegetation) that experience less disturbance and support higher plant diversity, are expected to sustain greater diversity and richness compared to field interiors. Given the relatively low spider captures during the first 2 years of the study, a second objective aimed to evaluate how pitfall trap characteristics including trap diameter, preservative substrate (propylene glycol vs. glass beads), and interior trap wall coating with a non‐stick fluoropolymer improve spider retention in grass and treed edges adjacent to canola fields.

## 2 Materials and Methods

### 2.1 Study Area

**FIGURE 1:**
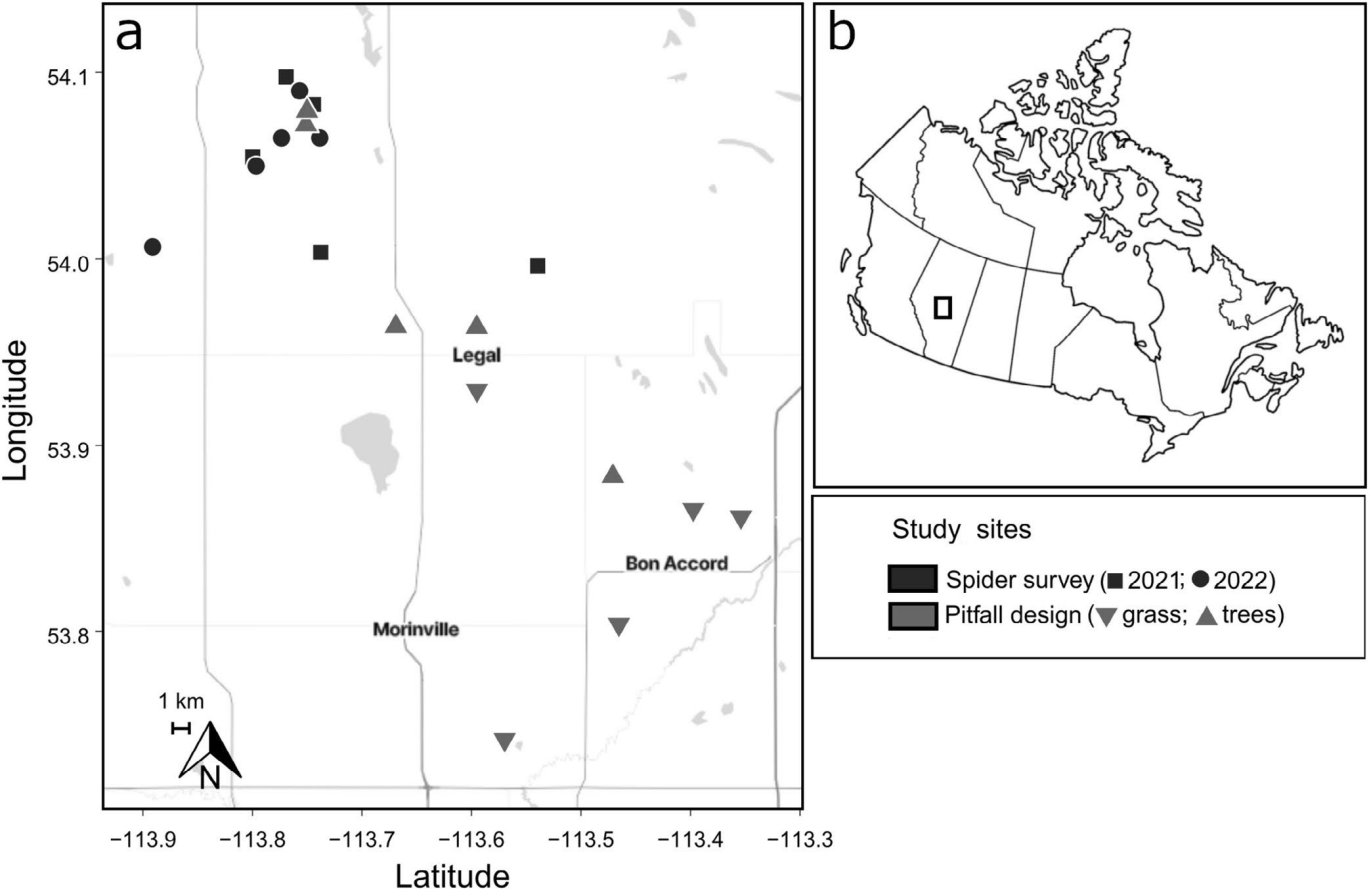
Locations of canola study sites in central Alberta, Canada, (a) used for sampling for spider biodiversity throughout the growing season in 2021 (black squares) and 2022 (black circles), and sites used in the pitfall trap design experiment in grassy (inverted triangle) and treed (triangle) canola field edges in 2023. (b) Inset map indicating study region within Canada.

Studies were conducted in rain‐fed, commercially managed spring‐planted canola fields located within the Aspen Parkland ecoregion, north of the city of Edmonton (113°29′ W, 53°32′ N), Alberta, Canada (Figure 1). This transitional zone, situated between the southern prairies and northern boreal forest (Young et al. 2006), is characterized by a mosaic of aspen ( *Populus tremuloides* Michx., Salicaceae) and white spruce ( *Picea glauca* (Moench) Voss, Pinaceae) stands interspersed with grasslands, much of which has been converted to agricultural land use (Bailey et al. 1990; Chapagain and Good 2015). The dominant soil types in the region are black and dark gray chernozems (AGRASID 2024). Canola field sizes ranged from 26 to 120 ha, with a minimum distance of 2 km between study sites to ensure spatial independence.

### 2.2 Spider Diversity and Composition in Canola

To assess spider diversity and activity density in canola fields, pitfall trapping was conducted between June and August 2021 (*n* = 5 sites) and 2022 (*n* = 5 sites) (Figure 1; Table 1). At each site, two parallel 100 m transects were established: one at a field edge, adjacent to semi‐natural non‐crop habitat (0.5 m from the edge), and another one at the field interior (100 m into the field) (Figure S1). The semi‐natural non‐crop habitat surrounding each field edge was dominated by herbaceous plants, grasses (Poaceae), and legumes (Fabaceae). Along each transect, five pitfall traps were installed at 25 m intervals. Traps were constructed with two 473‐mL plastic cups (8.5 cm diameter, 12.4 cm depth; Solo, ULINE, Canada, Milton, ON), one inside the other, with the outer cup perforated to allow drainage and the inner cup removable for easy servicing (Spence and Niemelä 1994; St Onge et al. 2018). Traps were installed flush with the soil surface and covered with a 15 × 15 cm corrugated plastic rain cover. This cover was placed approximately 5 cm above the trap and secured in all four corners using wooden dowels (Spence and Niemelä 1994; St Onge et al. 2018). Traps were filled with ~150 mL of propylene glycol (Plumbing Antifreeze, Certified, Canadian Tire, Edmonton, AB). Spiders (and other arthropods) were collected over three 7‐day collection periods each year (Table 1). Samples from all traps along each transect were pooled into a single 532 mL plastic bag (Whirl‐Pak, ULINE), stored on ice in insulated coolers for transportation to the laboratory, and then refrigerated at 4°C until processing.

**TABLE 1:** Total number of spiders captured in edge and interior transects in canola fields (*n* = 5 and 5, 2021 and 2022, respectively) in central Alberta, Canada.

Year	Date	Number of spiders		
		Edge transect	Interior transect	Total
2021	16–23 June	220	149	369
	21–28 July	100	21	121
	11–18 Aug	38	40	78
2022	16 June—23 June	97	90	187
	14–21 July	160	31	191
	18–25 Aug	56	15	71

*Note:* Edge transect was 0.5 m into the field; interior transect was 100 m into the field.

In the laboratory, spiders were sorted from other arthropods and stored in 70% ethanol. Spiders were identified to the species level under a dissection stereoscope (Nikon SMZ‐U) using relevant taxonomic literature (Dondale and Redner 1978, 1982, 1990; Platnick and Dondale 1992; Dondale et al. 2003; Paquin and Dupérré 2003). Spider taxonomy followed the World Spider Catalog V 25.0 (World Spider Catalog 2024). Unidentified and juvenile specimens were retained for family‐level analyses but omitted from species‐level analyses (see Section 2.4).

### 2.3 Pitfall Trap Design Comparisons

Due to the relatively low abundance and diversity of spiders collected in 2021 and 2022 (see Section 3), an experiment was conducted in 2023 to optimize the pitfall trap design for improved spider retention. The experiment evaluated trap diameter, preservation method (i.e., killing agent vs. live trapping), interior wall non‐stick coating (polytetrafluoroethylene, PTFE), and collection period length.

The experiment was conducted adjacent to 10 canola fields between June and August 2023 (Figure 1, Table 2). Two collection period durations, 24 h (*n* = 5) or 7 days (*n* = 3), were replicated throughout the growing season (Table 2). The two durations were selected to assess spider retention using two preservation methods: (1) wet traps with propylene glycol (24 h and 7 day), and (2) dry traps with 6 mm glass beads for live capture (24 h only). Short sampling durations with live traps are often required when specimens are needed for laboratory bioassays or downstream molecular assays, whereas longer sampling durations with wet traps are typically used to assess spider diversity and abundance. The traps were installed as described above.

**TABLE 2:** Total spiders captured combined across all experimental pitfall designs in the 24‐h and 7‐day collection periods in 2023. Pitfall traps were placed adjacent to canola fields next to grass or treed edges.

Collection period length	Date	Number of spiders		
		Grass edge	Tree edge	Total
24‐h	31 May—1 June	123	64	187
	21–22 June	252	94	346
	5–6 July	208	59	267
	19–20 July	112	61	173
	10–11 Aug	45	57	102
7‐day	24–31 May	722	836	1558
	12–19 July	288	243	531
	3–10 Aug	76	110	186

Three trap diameters, based on other studies (Brown and Matthews 2016; Hohbein and Conway 2018), were tested: large (14.5 cm diameter, 16.5 cm depth, 86.5° wall slope, 2.25‐L pail, Bee Maid Supplies, Spruce Grove, AB); medium (11.5 cm diameter, 7.6 cm depth, 80.7° wall slope, 473 mL deli container, ULINE); and small (8.5 cm diameter, 12.4 cm depth, 81.7° wall slope, 473 mL Solo cup, ULINE). Corrugated plastic rain guards were 4 cm larger than the corresponding trap diameter. To test if PTFE (ByFormica Fluon, Canada Ant Colony, Keswick, ON) coating increased spider retention, traps were either coated on the interior surface and rim or left untreated as controls. The coated area and preservative volume differed among trap sizes due to differences in depth (Table S1). All combinations of trap size and PTFE treatment were represented over the two studies.

Pitfall traps were set in a randomized complete block design (block = field site) across 10 canola fields, 5 with uncultivated weedy‐grass edges, and 5 with treed edges to sample a broader diversity of spiders. At each site, 12 traps were spaced 25 m apart along a 250 m transect parallel to and approximately 2.5 m from the first seed row outside the field (Figure S2). Transects in grass‐edged fields began > 20 m from neighboring field corners. These edges were primarily composed of fescue (*Festuca* spp.), dandelions (*Taraxacum* spp.), and occasional horsetail (*Equisetum* spp.). Treed‐edge transects began > 20 m away from the start of the tree line, where the tree line was either bisected by a road or met by a grass edge. Treed edges were dominated by cereal grasses (*Elymus* spp.) and trembling aspen. Vegetation was identified using regional field guides (Farrar 1995; Government of Alberta 2013; LaForge 2018).

At the end of each collection period, samples were retrieved from each trap separately. For traps containing propylene glycol, samples were strained using fine mesh bags to collect arthropods, and the residual liquid was discarded. For 24‐h live traps containing glass beads, the contents were carefully emptied into plastic bags (1 L) to minimize specimen damage. All samples were stored on ice for transport to the laboratory, where they were refrigerated at 4°C until processing. Live samples from glass bead‐containing traps were processed immediately by gently emptying the bag contents into water‐filled bins and hand‐collecting the spiders. Spiders from propylene glycol traps were separated by emptying the mesh bags into water‐filled trays and hand‐collecting the spiders. All spiders were preserved in 70% ethanol and identified to the species level as described above. Unidentified and juvenile specimens were retained for family‐level analyses but omitted from species‐level analyses (see Section 2.3). Voucher specimens are deposited in the Northern Forestry Center Arthropod Collection (Natural Resources Canada, Canadian Forest Service, Edmonton, Alberta).

### 2.4 Statistical Analyses

All statistical analyses were performed using relevant packages in R v. 4.3.1 (R Core Team 2023), as described below. Figures were generated using the R package *Esquisse* v. 1.1.2 (Meyer et al. 2024), and readability adjustments were implemented using Affinity Designer v. 2.4 (Serif Europe Ltd. 2022).

### 2.5 Spider Diversity and Composition in Canola

Coverage‐based rarefaction (Chao and Jost 2012) was used to estimate spider diversity over the sampling periods within the edge (0.5 m transect) and interior (100 m transect) of the crop (2021–2022), and among trap designs in each habitat (2023). In contrast to individual‐based rarefaction, in which estimations are compared under a minimum sample size (i.e., sample with the lowest number of individuals), coverage‐based rarefaction uses a minimum sampling coverage level (i.e., sample with the lowest coverage) for comparisons, which makes a more robust approach (Jost 2006, 2007). Within each year, transect data (interior or edge) were pooled across sites and sampling periods. Hill numbers (q0 = richness, q1 = exponential of Shannon's index, and q2 = inverse of Simpson's index) and coverage scores were obtained using *iNext* v.3.0 (Hsieh et al. 2022). For 2021–2022, data from interior and edge habitats were rarefied to a sample coverage of 94%. For 2023, data from the 24‐h and 7‐day sampling periods were rarefied to a sample coverage of 83% and 98%, respectively.

### 2.6 Pitfall Trap Design Comparisons

To test if pitfall trap size, PTFE treatment, and/or trap substrate influenced the retention rate of spiders, the number of spiders was assessed by fitting a Generalized Additive Model for Location, Scale, and Shape (*GAMLSS* v.5.4‐20) (Ziel 2022). A global model was constructed using pitfall catch data from all families to serve as test data for the initial model structure. This model included edge type (i.e., grass vs. treed), interior wall coating (i.e., PTFE vs. no PTFE), trap substrate (i.e., propylene glycol vs. glass beads), and trap size as fixed effects, and trap location within transect as random effects. The collection date were not included as a variable as it did not improve the accuracy of the model, and thus, data were pooled across collections. The following steps were employed for the creation of the global model, and subsequent models were used for assessing retained abundance of specific families. After initial model selection in *Glmulti* v1.08 (Calcagno 2020), the model was secondarily assessed using *GAMLSS*, which supported treating trap location as a potential source of autocorrelation, and a wider breadth of distribution families not supported by other generalized linear models, which ensured finding the best possible distribution family for assessing the data. Due to the low abundance of most families, targeted models were fit only for two (Lycosidae and Linyphiidae) of the 17 families represented in this study. The global model is not reported in the results as it was used for testing the model structure.

In the 24‐h collection experiment, *Glmulti* was used to initially select a model for assessing the impact of different trap variables. Trap treatment variables (size, substrate, and interior wall coating) were treated as fixed effects, as the generic screening algorithm found that the inclusion of interaction effects negatively impacted the model fit. After initial Akaike Information Criterion (AIC) based model selection in *Glmulti*, *GAMLSS* was used for a more extensive assessment and final build of all models. *GAMLSS* was used to ensure that the best distribution family was selected, using the *chooseDist* function and examination of the randomized quantile residuals. Both models used the Delaporte distribution family. Delaporte is a mathematical hybrid of the negative binomial and poisson distributions, which are both appropriate for assessing count data (Adler 2024). The *GAMLSS* modeling package was designed to be effective with small data sets, support the best‐fitting distribution family, and support the inclusion of correlation structures (Ziel 2022). *GAMLSS* provides model‐assessment metrics that are suggested to be more accurate than AIC, including quantile residuals of the mean (0) and variance (1), coefficient of skewness (−0.5 to 0.5), coefficient of kurtosis (3), and filliben correlation coefficient (1) (Groeneveld and Meeden 1984; Dunn and Smyth 1996; Hohberg et al. 2020). Following the scoring principles of Hohberg et al. (2020), the final *GAMLSS* model‐assessment for each of the families was based on the following metrics. Linyphiidae: mean = −0.056, variance = 0.98, coef. of skewness = 0.204, coef. of kurtosis = 3.07, filliben correlation coefficient = 0.998. Lycosidae: mean = 0.06, variance = 0.86, coef. of skewness = 0.054, coef. of kurtosis = 3.37, filliben correlation coefficient = 0.998.

*Parameters* v.0.21.3 (Lüdecke et al. 2020) was used to test post hoc multiple comparisons for significant main effects using Bonferroni correction to adjust *p*‐values. The *Parameters* package also provides standard error and confidence intervals. A forest plot of the response coefficients was created using *sjPlot* v.2.8.15 (Lüdecke et al. 2023).

## 3 Results

### 3.1 Species Diversity and Composition in Canola

During the 7‐day long sampling periods, in 2021 and 2022, a total of 568 and 400 spiders, respectively, were captured from all sites, represented by 74 spider species in 14 families (Table 1; Table S2). In 2021, the number of spiders captured peaked during the first collection period in June (Table 1). Spider abundance was lowest in the field interior, particularly during mid‐July each year (Table 1). The most abundant families across both years were Linyphiidae and Lycosidae (Table S2). A majority of Linyphiidae (131) were captured in the field interior compared to the field edge (65). Theridiidae (interior = 21, edge = 5) followed a similar trend. The opposite trend was observed for Lycosidae (interior = 146, edge = 436), Thomisidae (interior = 27, edge = 36), and Gnaphosidae (interior = 5, edge = 52) (Table S2). Eight of the remaining 10 families (Tetragnathidae, Phrurolithidae, Philodromidae, Hahniidae, Clubionidae, Titanoecidae, Agelenidae, and Liocranidae) were represented by less than 20 individuals each, with most of their capture occurring along the field edge. The two remaining families (Amaurobiidae and Araneidae) were represented by a single individual each in the field interior.

**FIGURE 2:**
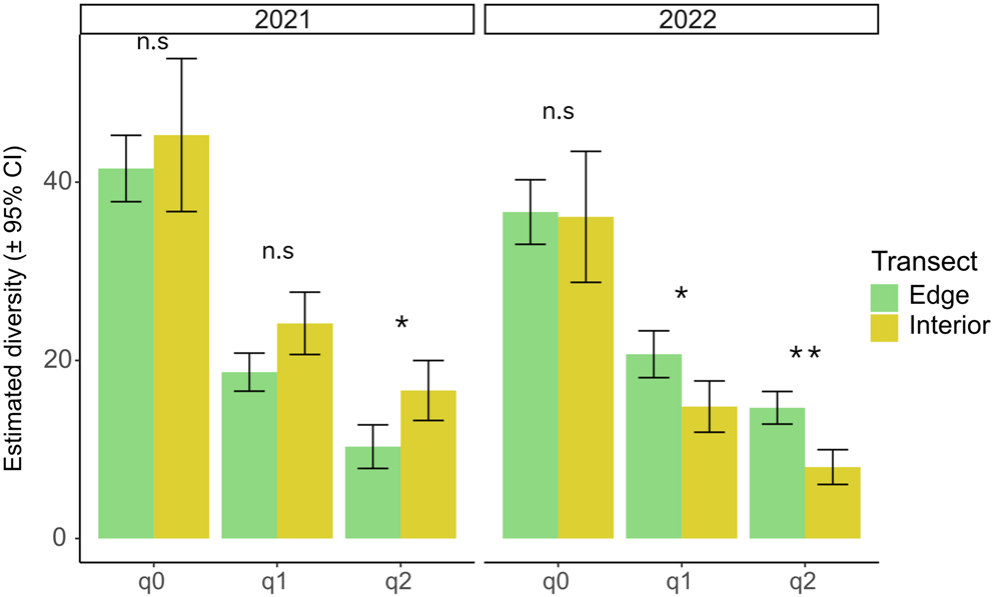
Estimated spider diversity in canola habitats in 2021 and 2022. Values for the different diversity orders (q0, q1, q2) were obtained using coverage‐based rarefaction (Chao and Jost 2012) with a sample coverage of 94% (n.s. = not significant; *p < 0.05; **p < 0.01).

There were no significant differences in estimated spider richness (q0) between the field interior and edge in each year (Figure 2). However, significant differences were observed in both years for Shannon's equivalent (q1; marginal in 2021) and Simpson's equivalent (q2) measures, with the field interior showing higher values in 2021 and lower values in 2022.

### 3.2 Pitfall Trap Design Comparisons

Across the five 24‐h collection periods, 854 spiders were collected, represented by 62 species in 12 families (Table 2; Table S3). While 42 species were collected from treed edges, 37 were captured from grassy edges. There were 17 species shared between the two habitats, 16 exclusive to the former, and 13 to the latter. With respect to trap treatments, 12 species were collected only in PTFE‐treated traps and six only in untreated traps from treed edges. In contrast, in grassy edges, 5 and 6 unique species were collected in PTFE‐treated and untreated traps, respectively (Table S3).

**FIGURE 3:**
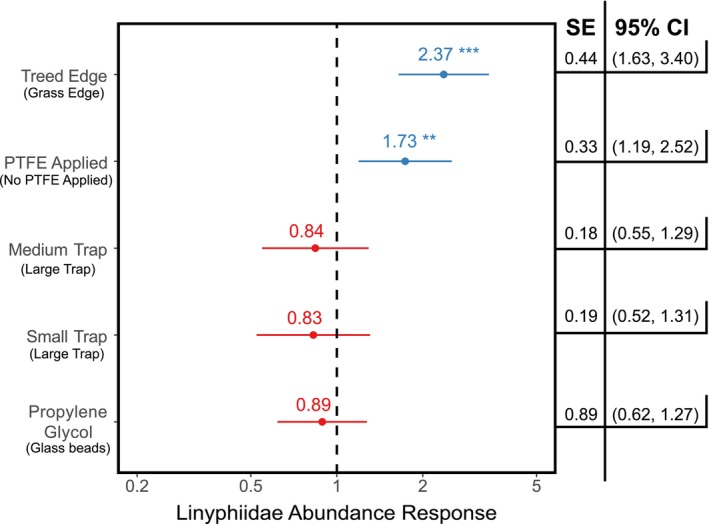
Abundance responses in Linyphiidae to trap treatments in 24‐h collection periods. Points are the mean abundance by treatment type and whiskers the 95% confidence interval (red indicates negative response; blue indicates positive response; **p < 0.01; ***p < 0.001). Treatment levels overlapping with the dashed vertical line denote no response with respect to the alternative (i.e., propylene glycol is compared against glass beads; small and medium trap sizes compared against large traps; PTFE applied is compared against no PTFE; treed edge compared against grass edge).

**FIGURE 4:**
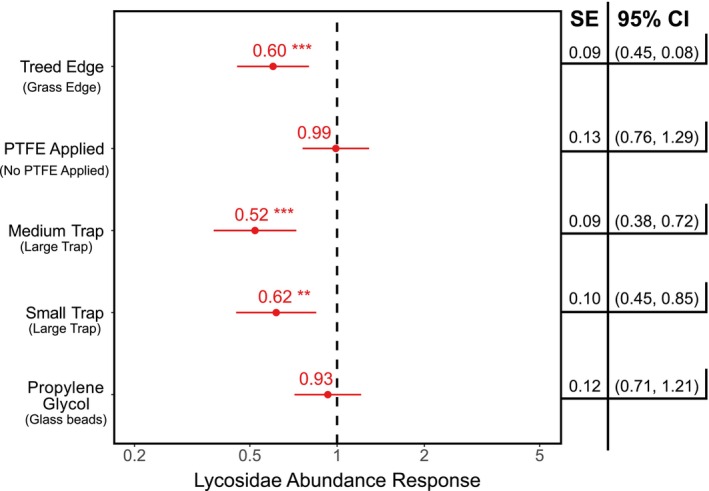
Abundance responses in Lycosidae to trap treatments in 24‐h collection periods. Points are the mean abundance by treatment type and whiskers the 95% confidence interval (red indicates negative response; blue indicates positive response; **p < 0.01; ***p < 0.001). Treatment levels overlapping with the dashed vertical line denote no response with respect to the alternative (i.e., propylene glycol is compared against glass beads; small and medium trap sizes compared against large traps; PTFE applied is compared against no PTFE; treed edge compared against grass edge).

The *GAMLSS* model detected significant combined effects of habitat, trap diameter, PTFE treatment, and preservation substrate for both Linyphiidae (*t*_(272)_ = −5.22, SE = 0.07, *p* = < 0.001) and Lycosidae (*t*_(272)_ = 7.08, SE = 0.47, *p* = < 0.001). The abundance of linyphiids increased in response to treed field edges (response coefficient = 2.37, SE = 0.44, *t*_(272)_ = 4.66, *p* < 0.001) and with the application of PTFE (response coefficient = 1.73, SE = 0.33, *t*_(272)_ = 2.89, *p* = 0.004) (Figure 3). However, no significant effects were detected in relation to trap diameter (averaged response coefficient and SE for small and medium traps: response coefficient = 0.835, SE = 0.185) or trap preservation substrate (response coefficient = 0.89, SE = 0.89). The abundance of Lycosidae was not affected by the application of PTFE or preservation substrate, but there was a significant effect with respect to habitat, with lower abundances in treed edges (response coefficient = 0.60, SE = 0.09, *t*_(272)_ = −3.51, *p* < 0.001) (Figure 4). Similarly, a significant effect with respect to trap diameter was detected, with lower abundances in small (response coefficient = 0.62, SE = 0.10, *t*_(272)_ = −3.00, *p* = 0.003) and medium traps (response coefficient = 0.52, SE = 0 0.09, *t*_(272)_ = −3.92, *p* < 0.001) (Figure 4).

The application of PTFE combined with glass beads (trap preservation substrate) significantly increased the diversity of spiders captured in the large traps in treed environments (q0: qD = 28.2, CI = 8.1, q1: qD = 26.9, CI = 8.6, qD = 24.8, CI = 8.6), compared to untreated large dry traps (q0: qD = 10.25, CI = 5.70, q1: qD = 8.27, CI = 3.99) (Figure S3). No significant effects, however, were observed in the grass habitat or the smaller trap sizes (Figure S3).

During the 7‐day collection periods, 1587 spiders were collected, represented by 72 species in 16 families (Table 2; Table S4). While 56 species were captured in the treed habitat along the edge of canola fields, 25 species were captured in the grass habitats. There were 26 species exclusive to the former and 15 species to the latter. While one species ( *Pardosa moesta* ) was caught in all habitats, trap treatments, and trap diameters, nine were captured in most and were only absent from one or two trap treatment‐size‐habitat combinations. Moreover, 27 species were captured by only one trap type across both habitats. Four species were captured only in non‐PTFE traps in the grass habitats. Five species were captured in only the PTFE‐treated traps in the grass habitats, whereas seven species were captured in only the non‐PTFE traps in the tree habitats, and 13 species were captured only in the PTFE‐treated traps in the treed habitats (Table S4).

**FIGURE 5:**
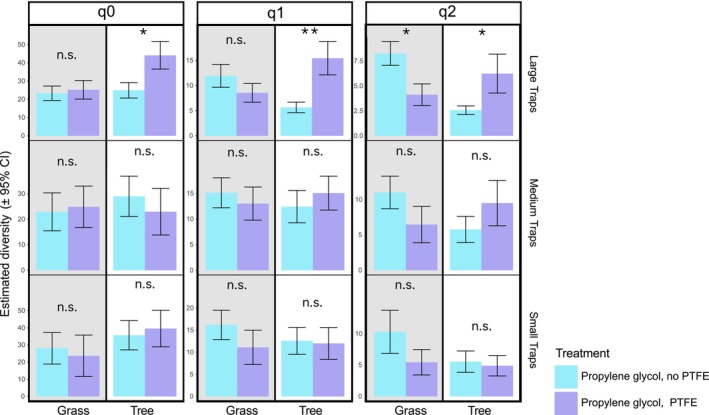
Estimated diversity (q0, q1, and q2) in a 7‐day collection period by trap size, type, and location. All treatment combinations were rarefied to a sample coverage of 98% (n.s. = not significant; *p < 0.05; **p < 0.01). Gray background represents the grass collection sites; white backgrounds are treed collection sites.

While PTFE application significantly increased the estimated diversity (q0, q1, and q2 diversity orders) of spiders captured in large traps in the treed environments, it significantly reduced q2 diversity in the grass environment (Figure 5). In both medium and small traps, PTFE had no significant effect in either habitat for any diversity order, except for a marginally lower effect in grass habitats (Figure 5).

## 4 Discussion

This study assessed the diversity and composition of ground‐dwelling spider assemblages in canola agroecosystems in the Aspen Parkland region of Alberta, Canada, documenting 119 spider species from 17 families over 3 years (Tables S2 and S3). Similar to other temperate agroecosystems globally, Lycosidae and Linyphiidae dominated the assemblage (e.g., Sunderland and Samu 2000; Nyffeler and Sunderland 2003). Similar patterns were previously reported in undisturbed forests and tall grass prairies of Alberta and Manitoba (Buddle and Shorthouse 2008; Roughley et al. 2006), although this study is among the first to focus on canola systems. While more than 800 spider species are known from Canadian grasslands (Cárcamo et al. 2014), the subset collected here likely reflects both habitat characteristics and sampling constraints. Nonetheless, the detection of 119 species highlights the ecological resilience of epigaeic spider assemblages in intensively cultivated agroecosystems.

Spider abundance and diversity varied with field position. Diversity was higher in field interiors in 2021 but lower in 2022 possibly due to interannual differences in climate, crop growth, or edge vegetation structure. The lack of a significant effect on spider diversity may also reflect the limited sampling effort, with only three 7‐day collection periods per year, potentially reducing overall species capture, particularly rare taxa. Lycosidae, especially *Pardosa distincta* and *P. moesta* , were more abundant at field edges, whereas Linyphiidae and Theridiidae were more common in field interiors. These spatial patterns likely reflect species‐specific microhabitat preferences, hunting strategies, and tolerance to abiotic factors such as temperature and humidity (Langellotto and Denno 2004). For instance, *P. moesta* is known to migrate into canola fields in early summer and disperse later in the season, while *P. distincta* appears more resident (Robinson et al. 2021). Field‐edge traps likely captured these seasonal shifts in activity. In contrast, Linyphiidae were more common in field interiors, possibly due to their affinity for humid microhabitats fostered by dense canola canopies (Pearce et al. 2004; Pluess et al. 2010). Intra‐guild competition may also structure these communities, reflecting observed differences in spider diversity and abundance. Larger lycosids may be limited in the interior due to prey competition, while smaller prey populations may sustain higher linyphiid abundance (Uetz 1977; Rusch et al. 2015). These findings are consistent with several other studies in agricultural and forest systems that sampled spiders using pitfall traps and observed similar patterns, with dominance of lycosids complemented by linyphiids that were present in smaller but still notable numbers (Huhta 1965; Doane and Dondale 1979; Ferguson et al. 1984; Djoudi et al. 2018).

Several species were identified as indicators of field edge habitats (Table S5), consistent with patterns in other agroecosystems (Samu and Szinetar 2002; Schmidt and Tscharntke 2005). In contrast, the field interior supported fewer individuals and a less diverse spider community, with no identified indicator species, likely due to habitat homogeneity and greater disturbance, which may support fewer specialists (Table S6). These results emphasize the ecological value of semi‐natural edges in maintaining spider biodiversity in agricultural systems (Schmidt and Tscharntke 2005; Samaranayake and Costamagna 2018).

This is particularly relevant for potential biological control of canola pests, as field edges can harbor predators and may also buffer pest incursions into the fields (Samaranayake and Costamagna 2018; Maino et al. 2019; Crowther et al. 2023). Many species observed in this study (e.g., *P. moesta* and *P. distincta* ) are known to feed on key pest species (Nyffeler and Sunderland 2003; Kuusk and Ekbom 2012; Robinson et al. 2021), including aphids and flea beetles (Amjad et al. 2017; Ekbom et al. 2014). While edge‐dwelling spiders may reduce pest ingress into fields, their limited presence in field interiors may restrict their biocontrol potential (Kuusk and Ekbom 2012; Robinson et al. 2021). Enhancing habitat quality and complexity at field margins could improve spider‐mediated control and reduce reliance on insecticides. Notably, increasing semi‐natural vegetation does not necessarily reduce yield and may even enhance productivity (Galpern et al. 2020). These findings are in line with those from Europe and Australasia, where spider conservation is an integral part of conservation biological control (Ekbom et al. 2014; Benamú et al. 2017). Dominant species such as *P. moesta* and *P. distincta* represent promising targets for future predator–prey studies, but their efficacy will depend on spatial and temporal overlap with pest populations (Glück and Ingrisch 1990; Birkhofer et al. 2008).

Pitfall trap characteristics significantly influenced capture rates and diversity estimates. Traps used in 2021 and 2022 may have been suboptimal due to their smaller diameter and the presence of interior ridges, which can facilitate spider escape or reduce retention (Brennan et al. 1999; Work et al. 2002; Lange et al. 2011). This is the first study to test the effects of PTFE coatings on spider capture in pitfall traps, despite being employed in other studies (Koponen et al. 1997; Lucey and Hill 2012). Our trap modification experiment showed that trap diameter, PTFE treatment, and substrate type significantly affected spider captures. Larger traps treated with PTFE improved spider retention and increased overall abundance and diversity, particularly in treed‐edge habitats. PTFE likely reduced escape, particularly for linyphiids, via silk adhesion or climbing ability (Grawe et al. 2014). Although lycosids were less affected by PTFE, overall collection efficiency improved with trap modifications, especially for taxa sensitive to traditional trap designs (Work et al. 2002; Lange et al. 2011). Although PTFE application can increase workload and pitfall designs are numerous, this approach provides clear benefits for the targeted live collection of ground‐dwelling spiders (Brown and Matthews 2016). In these cases, and in studies looking to establish baseline diversity in a system or ensure maximal representation of ground‐dwelling spider diversity, we recommend the use of both PTFE coating and sufficiently large‐sized traps.

Surprisingly, ‘dry’ pitfall traps with glass beads retained more spiders than traps with propylene glycol during the 24‐h trials, contrary to previous studies (Table S3, Figure S3) (Weeks Jr. and McIntyre 1997; Winder et al. 2001; Schmidt et al. 2006). The resulting increase in the retention of live spiders supports many research avenues, such as genetic, ontogenetic, behavioral, and conservation work. While it was not explicitly tested, the glass beads in dry traps were used over plant material and soil, clay balls, or a variety of other substrates that have been used by other researchers (e.g., Staudacher et al. 2018). Beads create more consistent and uniform gaps for small spiders to hide from larger predators. They also abrade the walls of traps less, increasing reusability and ease of cleaning, as well as providing ease of visibility for researchers retrieving specimens. While survival within bead traps was not quantified, flooding the traps in the laboratory easily yielded viable specimens for live‐based assays.

While this study enhances our understanding of spider diversity and trap efficiency in canola fields, several limitations should be acknowledged. Juvenile spiders, which were common in samples, were excluded from species‐level analyses, potentially underestimating species richness. Additionally, pitfall traps may not capture the full range of spider activity or vertical stratification, especially for arboreal or web‐building species. Future research should integrate complementary sampling methods (e.g., sweep netting and suction sampling) and evaluate the functional roles of spiders in pest control through gut content analysis or direct observation.

This study provides baseline data on spider assemblages in canola agroecosystems and demonstrates the value of pitfall trap refinement for improving passive sampling. These findings highlight the ecological role of field edges, the importance of habitat‐specific community responses, and the potential of certain species as biocontrol agents or indicators. These insights can inform conservation biological control strategies and contribute to more sustainable pest management in Prairie cropping systems.

## Author Contributions

**Kirra Kent:** conceptualization (equal), data curation (lead), formal analysis (lead), investigation (lead), methodology (equal), visualization (lead), writing – original draft (lead), writing – review and editing (equal). **Aldo Ríos Martínez:** conceptualization (equal), investigation (equal), methodology (equal), writing – review and editing (equal). **Kristen Guelly:** investigation (equal), validation (equal), writing – review and editing (equal). **Jaime Pinzon:** conceptualization (equal), methodology (equal), supervision (equal), writing – review and editing (equal). **Boyd A. Mori:** conceptualization (equal), funding acquisition (lead), methodology (equal), project administration (lead), resources (lead), supervision (lead), writing – review and editing (equal).

## Conflicts of Interest

The authors declare no conflicts of interest.

## Supporting information


**Figures S1–S3:** ece372205‐sup‐0001‐FiguresS1‐S3.docx.

**Tables S1–S6:** ece372205‐sup‐0002‐TablesS1‐S6.xlsx.

## Data Availability

All relevant data are included within the Supporting Information, and the complete dataset has been deposited in Dryad (https://datadryad.org/share/10.5061/dryad.34tmpg4xh) Code for the analyses is available from the corresponding author(s) upon reasonable request.
