# Supplementary material for: Edge Effects and Pitfall Trap Design Influence Spider Diversity and Assemblages in Canola Agroecosystems on the Canadian Prairies
Source: Ecol Evol. 2025 Sep 23;15(9):e72205. doi: 10.1002/ece3.72205 (PMC12457220; doi:10.1002/ece3.72205)
Supplement: Supplementary file 1 — Figures S1–S3: ece372205‐sup‐0001‐FiguresS1‐S3.docx. [file ECE3-15-e72205-s001.docx]

#### **Supplemental Methods:**

#### Indicator Species Analysis:

To assess if any species were significantly associated with either the field interior or edge habitats (2021-2022), indicator species analysis was performed by using *Indicspecies* v.1.7.14 (De Cáceres & Legendre, 2009). Significance (p < 0.05) of indicator values was assessed through 999 permutations. For 2023, a similar approach as above was taken to estimate diversity and identify indicator species as a function of trap types and habitats (grass vs. treed edges) over time for both 24-hour and 7-day collection periods.

**Supplemental Results:**

Indicator Species Analysis:

*Spider diversity and composition in canola*

Seven species were found to be significant indicators of canola field edges: *Pardosa distincta* (Blackwall, 1846), *Pardosa moesta* Banks, 1892, *Trochosa terricola* Thorell, 1856, *Micaria pulicaria* (Sundevall, 1831), *Pardosa ontariensis* Gertsch, 1933, *Micaria rossica* Thorell, 1875, *Pardosa modica* (Blackwall, 1846) (Supp. Table 5). No significant indicator species were detected for the field interior.

*Pitfall trap design comparisons*

Eight species were significant indicators of either treed and grassy habitats (Suppl. Table 6), accounting for 66.3% of the total spiders in the 7-day collection period experiment, with 29.5% represented by *P. moesta* and 14.1% by *P. distincta* (Supp. Table 4 & 6).

**Supplemental Figures:**

#### **
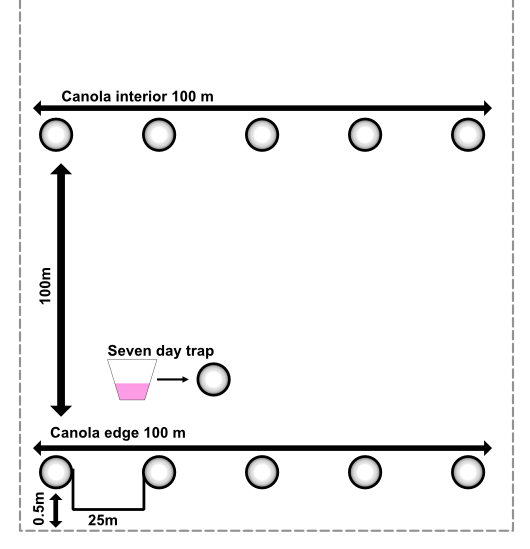
**

**Supplemental Figure 1.** Experimental design for the 2021-2022 diversity study. Light circles represent pitfall traps that were opened seven days per month. Canola field edge represented by dashed grey line. All traps were 8.5 cm in diameter, 12.4 cm deep.

#### **
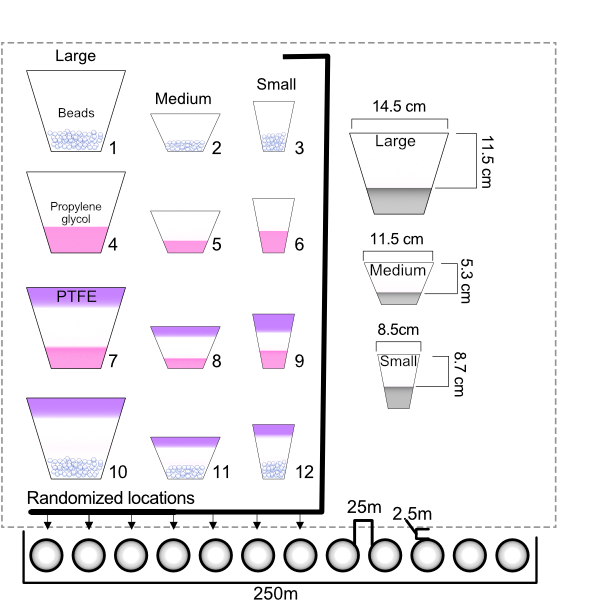
**

**Supplemental Figure 2.** Experimental design for the 2023 trapping methods study. Canola field edge represented by a dashed line. Depth to substrate shown for all trap sizes, fill volume of both substrates was between 25-30% for all traps.


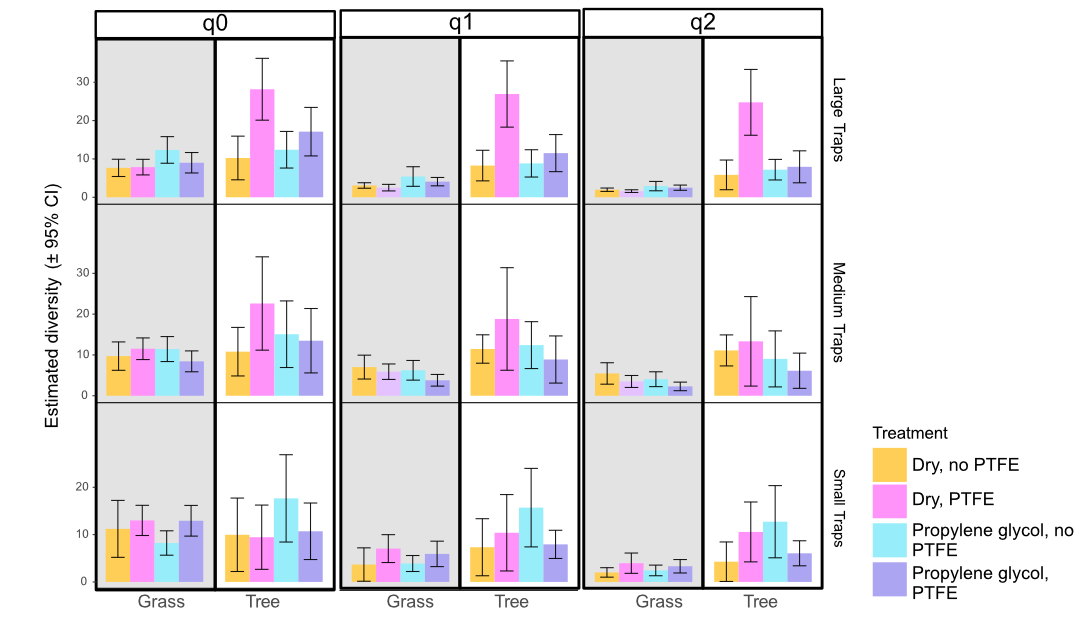


**Supplemental Figure 3.** Estimated diversity in 24-hour collection period by trap size, PTFE and substrate treatment, and habitat type. All samples were rarefied to a sample coverage estimated to be 82%.
